# Replicating the Violence Risk Appraisal Guide: A Total Forensic Cohort Study

**DOI:** 10.1371/journal.pone.0091845

**Published:** 2014-03-14

**Authors:** Astrid Rossegger, Jérôme Endrass, Juliane Gerth, Jay P. Singh

**Affiliations:** 1 Department of Mental Health Services, Office of Corrections, Canton of Zurich, Zurich, Switzerland; 2 Department of Psychology, University of Konstanz, Konstanz, Germany; 3 Institute of Health Sciences, Molde University College, Molde, Norway; The University of Queensland, Australia

## Abstract

**Introduction:**

The performance of violence risk assessment instruments can be primarily investigated by analysing two psychometric properties: discrimination and calibration. Although many studies have examined the discrimination capacity of the Violence Risk Appraisal Guide (VRAG) and other actuarial risk assessment tools, few have evaluated how well calibrated these instruments are. The aim of the present investigation was to replicate the development study of the VRAG in Europe including measurements of discrimination and calibration.

**Method:**

Using a prospective study design, we assessed a total cohort of violent offenders in the Zurich Canton of Switzerland using the VRAG prior to discharge from prisons, secure facilities, and outpatient clinics. Assessors adhered strictly to the assessment protocol set out in the instrument’s manual. After controlling for attrition, 206 offenders were followed in the community for a fixed period of 7 years. We used charges and convictions for subsequent violent offenses as the outcomes. Receiver operating characteristic analysis was conducted to measure discrimination, and Sanders’ decomposition of the Brier score as well as Bayesian credible intervals were calculated to measure calibration.

**Results:**

The discrimination of the VRAG’s risk bins was modest (area under the curve = 0.72, 95% CI = 0.63–0.81, *p*<0.05). However, the calibration of the tool was poor, with Sanders’ calibration score suggesting an average assessment error of 21% in the probabilistic estimates associated with each bin. The Bayesian credible intervals revealed that in five out of nine risk bins the intervals did not contain the expected risk rates.

**Discussion:**

Measurement of the calibration validity of risk assessment instruments needs to be improved, as has been done with respect to discrimination. Additional replication studies that focus on the calibration of actuarial risk assessment instruments are needed. Meanwhile, we recommend caution when using the VRAG probabilistic risk estimates in practice.

## Introduction

Many actuarial risk assessment instruments have been developed over the past 30 years in response to seminal research [1], [2] and government reports [3]–[5] on the poor predictive validity of unstructured clinical judgments regarding the prediction of violence. These structured instruments are composed of weighted risk and protective factors that have been found to be statistically associated with the likelihood of violence. To obtain an estimate of risk, one combines the factors using a pre-determined algorithm that assigns subjects to a risk category or “bin” to which the instrument’s creators have assigned an empirically determined probability of future violence [6]. In doing so, these empirically developed risk assessment instruments offer a probability model rather than predicting one of two outcomes (recidivism vs. no recidivism; cf. [7], [8]).

According to recent surveys [9], [10], one of the actuarial instruments most commonly used by clinicians is the Violence Risk Appraisal Guide (VRAG; [11]). The VRAG was developed in Canada using a sample of 618 adult mentally disordered violent offenders at the Mental Health Centre in Penetanguishene [12]. The offenders were followed for 6.8 (*SD* = 5.1) years after discharge and both charges and convictions for subsequent violent offenses were identified. The scheme was constructed using the Nuffield [13] strategy, which identifies items and subsequently assigns weights according to how well the characteristics differentiate between the base rates of offending. Since its publication, the VRAG has become one of the best-researched instruments in terms of studies designed to measure its performance to assess the risk of recidivism [14].

Despite widespread implementation of the VRAG and its large research base, a systematic review suggests that no studies have been published that replicate the original development study of the VRAG in terms of sex and age composition, sample index offense, use of file information for administration, reliable scoring, lack of item approximations and omissions, length of (fixed) follow-up, controls for attrition, assessment of violent recidivism, and use of conviction as the legal status of outcomes [15]. Moreover, there are few studies attempting to replicate the probabilistic estimates put forth in the VRAG manual for the instrument’s nine actuarial risk bins (Table 1). Studies that *have* investigated the goodness-of-fit between the rates of violent recidivism published in the VRAG manual and rates observed during the research have produced inconsistent findings (Table 2). This inconsistency is important given the equal importance of discrimination and calibration when attempting to establish a valid risk assessment tool.

**Table 1:** Previous studies investigating calibration of the VRAG.

	Previous studies									Current study
	Harriset al. [12] 1	Tengström[47]	Harris et.al.[45]	Harris et al.[48]	Mill [49]	Yessineet al. [50]	Snowdenet al. [51]	Kröneret al. [52]	Hastings et. al.[53] 2	Zurich ForensicStudy
Replication match^3^	–	3	7	7	7	6	6	5	5	12
No item approximations	–	No	Yes	Yes	Yes	No	Yes	Yes	Yes	Yes
No systematic item omission	–	No	Yes	Yes	Yes	No	Yes	Yes	Yes	Yes
Reliable scoring	Yes	No	Yes	Yes	No	Yes	Yes	No	Yes	Yes
Controlling for attrition	Yes	No	No	No	No	Yes	No	Yes	No	Yes
File information used for scoring	Yes	Yes	Yes	Yes	Yes	Yes	Yes	Yes	No	Yes
LoFU (years)	6.8 (Mean)	7.2 (Mean)	7.1 (Mean)	5.1 (Mean)	NR	3.4 (Mean)	5.0 (Fixed)	4.8 (Mean)	1.0 (Fixed)	7.0 (Fixed)
Recidivism criteria	Charge+Conviction	Conviction	Charge	Charge	Charge	Conviction	Conviction	Conviction	Self-report	Charge+Conviction
Recidivism rate^4^	31%	29%	29%	48%	29%	48%	13%	19%	20%	17%

*Note.* – = Not Applicable; NR = Not Reported; LoFU = Length of follow-up.

1 VRAG development sample.

2 Rates for men only.

3 Out of 12 matching criteria established by Rossegger and colleagues [15].

4 Base rate of violent (including sexual) recidivism for offenders with a VRAG score.

**Table 2:** Previous studies investigating calibration of the VRAG with respect to recidivism rate.

	Previous studies									Current study
	Harris et al.[12] 1	Tengström[47]	Harris et.al.[45]	Harris et al.[48]	Mill [49]	Yessine et al.[50]	Snowden et al.[51]	Kröner et al.[52]	Hastings et. al.[53] 2	Zurich ForensicStudy (n)
VRAG risk bin	Recidvism rate									
1	0%	0%	17%	0%	0%	0%	0%	0%	0%	– (0)
2	8%	0%	11%	0%	0%	0%	0%	11%	0%	10% (1)
3	12%	15%	15%	20%	15%	0%	6%	27%	0%	0% (0)
4	17%	18%	21%	31%	18%	33%	6%	30%	5%	10% (4)
5	35%	29%	42%	39%	15%	22%	6%	33%	9%	15% (6)
6	44%	42%	58%	51%	33%	55%	23%	61%	17%	19% (8)
7	55%	46%	70%	65%	43%	73%	29%	80%	37%	32% (11)
8	76%	100%	71%	84%	48%	67%	44%	0%	75%	50% (4)
9	100%	0%	100%	89%	33%	100%	33%	100%	100%	43% (3)

*Note. n* = absolute numbers of recidivists; – = Not Applicable;

1 VRAG development sample.

2 Rates for men only.

### Components of Performance Measures

When thinking about the performance of a violence risk assessment instrument, two distinct aspects deserve attention – discrimination and calibration [16]. In the present context, *discrimination* refers to the instrument’s ability to differentiate between recidivists and non-recidivists, and *calibration* refers to the fit between the risk estimates provided by the instrument’s creators (which typically are based on the recidivism rates in the sample used to develop the tool) and the observed recidivism rates in the sample of current interest.

As several researchers have pointed out, discrimination findings for actuarial instruments do not necessarily equate to calibration validity (cf. [17]–[19]); rather, discrimination and calibration are equally important sides of the same coin, both of which need to be established in order to argue that a tool is valid [20]. Therefore, despite a considerable evidence base supporting the VRAG’s discrimination [21], evidence of the tool’s calibration (i.e., the ability of a risk assessment tool to estimate rates of recidivism for single risk scores) remains an essential piece of the puzzle that is currently incomplete.

### The Present Study

In the present investigation it was our aim to replicate the initial development study of the VRAG in Europe, paying particular attention to matching the demographic and design characteristics of the tool’s normative investigation. Both discrimination and calibration performance indicators were calculated. We hypothesised that discrimination and calibration indices would be satisfactory using a sample and methodology that did not differ substantially from the original VRAG study.

## Method

### Participants

**Figure 1:**
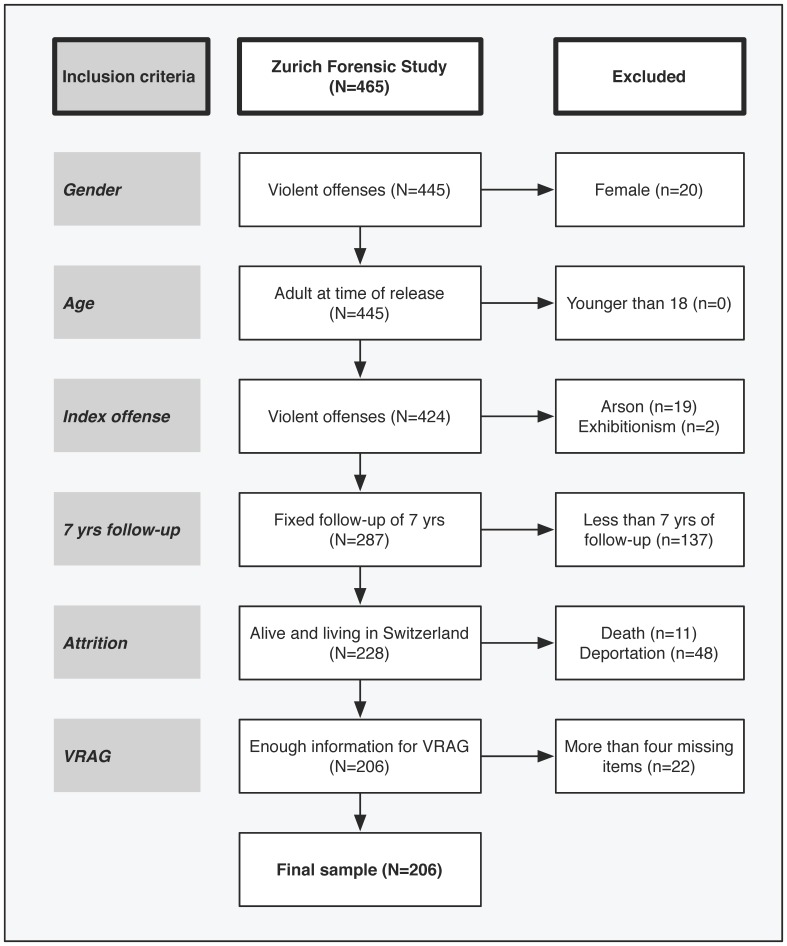
Sample selection process for the total forensic cohort from the Zurich Canton of Switzerland.

The sample for the present study was taken from the Zurich Forensic Study, a prospective study of all 465 offenders supervised by the criminal justice system of the Canton of Zurich, Switzerland, as of August 2000 [22]. This total forensic cohort included all offenders regardless of the severity of their index offense, mental health status, criminal responsibility, and length of prison stay provided a minimum sentence of 10 months or court-ordered therapy was carried out. To make the study sample comparable to the VRAG development sample, we considered only male offenders who were discharged into the community and who achieved a follow-up time of 7 years (*n* = 287). After elimination of participants who died, were deported before the end of the follow-up period, or were missing five or more VRAG items, the final study sample consisted of 206 offenders (Figure 1). Research using this dataset was approved by the Ethics Committee of the Canton of Zurich. With agreement from the committee, informed consent was not needed because there was no contact with any of the study participants.

### Procedure

The present study represents the first true replication study of the VRAG performed according to the comprehensive criteria for matching design and demographics that were established by Rossegger and colleagues [15] to compare validation and cross-validation studies (Table 3).

**Table 3:** Match of design and demographic characteristics of the present sample with that from the VRAG development study.

Replication criterion	Zurich Forensic Study	Match
Offender sex	Only males	Yes
Offender age	Only adults	Yes
Index offense	Violent and (hands-on) sex offenses	Yes
Using file information	Official files considered: criminal record, correctional and clinical files	Yes
Reliable scoring	Trained raters	Yes
No item approximations	No approximations	Yes
No systematic item omission	No omissions	Yes
Length of follow-up	7 years	Yes
Fixed length of follow-up		Yes
Controlling for attrition	Death, leaving jurisdiction, change of names	Yes
Type of recidivism	Violent (including sexual) recidivism	Yes
Legal status of recidivism	charges and convictions^1^	Yes

*Note.* Replication criteria are derived from the systematic review of Rossegger and colleagues [15].

1 In Switzerland, charges are only displayed in criminal records while a subject is under investigation.

Two masters-level psychologists who had attended accredited Psychopathy Checklist-Revised [23] workshops and were blind to the purpose of the study and participant outcomes scored a validated translation of the VRAG [24]. The assessors adhered strictly to the assessment protocol set out in the instrument’s manual, avoiding systematic item omissions and using the prorating algorithm published by the VRAG authors [11]. A pilot study revealed substantial inter-rater agreement between the item and total scores of the two assessors (κ = 0.70–0.89 [25]).

Recidivism was defined as a new charge or conviction for a violent (including sexual) offense committed after discharge from prisons, secure facilities, and outpatient clinics. Determination of recidivism was based on criminal records, which included information on charges and convictions, date of offense, type of offense, and length of sentence. Of note, in Switzerland charges are only displayed in the criminal record while a subject is under investigation. The potential time at risk was from August 2000 until May 2011. In order to create a follow-up period comparable to that used in the VRAG development study, we considered only offenses committed within 7 years after discharge.

### Statistical Analysis

Discrimination was measured using receiver operating characteristic (ROC) curve analysis and the resulting area under the curve (AUC). The ROC curve plots the true positive rate (the fraction of recidivists correctly identified) as a function of the false positive rate (the fraction of nonrecidivists misidentified) as the decision criterion (or cut-off) is moved from the highest to the lowest risk bin. The AUC represents the probability that a randomly selected recidivist would have a higher risk bin classification than a randomly selected non-recidivist.

Calibration was measured using three methods. First, we compared the violent recidivism rates for each VRAG risk bin during the 7-year fixed follow-up period in the development study published by Harris and colleagues [12] with the recidivism rates of participants in the total forensic cohort of this study. Second, we calculated the squared error between the average predicted recidivism rate and the average observed recidivism rate in each risk bin using Sanders’ decomposition of the modified Brier score [26]. The Brier score is a commonly known overall performance measure calculating the disagreement between expected rates and a binary variable (i.e. the mean squared error of prediction) [27]–[29]. Thus, it addresses both – the discrimination and calibration of a model and ranges from 0 to 1 with 0 suggesting a perfect model performance (cf. [30]). These properties (discrimination and calibration) can be analysed separately by using the Sanders’ decomposition of the modified Brier score. The first term of the Sanders’ decomposition of the modified Brier score provides information on the calibration as it measures the error that emerges from the mean forecast within the group without measuring the mean outcome within the group. The second term contains the discrimination of the model [30], [31]. An overview of the Brier score is provided by Ferro [32] and Redelmeier, Bloch, and Hickam [33]. Third, we calculated Bayesian credible intervals for the VRAG’s risk bin of the Zurich Forensic Study by using the Jeffreys’ prior for the Beta distribution [34]. We applied a Bayesian approach to investigate the observed data by comparing the bin-specific rates with those published by the tool’s authors considering a prior probabilistic distribution [35]–[37].

To conduct discrimination analyses we used the “roc” command and calibration analyses were conducted using the “brier” and “ciji” command in STATA/IC 12.1 for Windows and Mac [38]. For all analyses, we calculated two-tailed tests with a standard significance threshold of α = 0.05.

## Results

### Sample Characteristics

The sample population for the present investigation was composed of 206 adult male offenders with a mean age of 34.8 years (*SD* = 11.5) at the time of conviction for their index offense and 37.6 years (*SD* = 11.7) at their discharge. Index offenses included the following: homicide (*n = *37, 18.0%), robbery (*n* = 55, 26.7%), assault (*n* = 31, 15.1%), child sexual abuse (*n* = 44, 21.4%), and rape (n = 39, 18.9%). Court-mandated therapy was ordered for 131 (63.6%) offenders. Criteria for a personality disorder according to DSM-IV and/or ICD-10 were fulfilled in 45.6% (*n* = 94) of the offenders, and 11.1% (*n* = 23) of the sample met the diagnostic criteria for schizophrenia.

### Base rate of Violent Recidivism

The cohort was followed for 7 years post-discharge and criminal registers were used to ascertain whether they had recidivated or not. The base rate of violent (including sexual) recidivism was 18.0% (*n = *37). When stratified by offense type, the following recidivism rates were documented: homicide, 1.5% (*n* = 3); robbery, 5.3% (*n* = 11); assault, 8.7% (*n* = 18); child sexual abuse, 3.9% (*n* = 8); and rape, 1.9% (*n* = 4). Three participants engaged in acts that were classified under more than one category.

### Performance Measures

The discrimination analysis of the VRAG – assessed by using ROC curve analysis – produced an AUC of 0.72 (95% CI = 0.63–0.81, *p*<0.05). This suggests that the probability that a randomly selected violent recidivist had a higher risk bin classification than a randomly selected non-recidivist was 72%. Although there is considerable variability in what constitutes a small, moderate, and large value for AUC [39], there is general agreement that this effect size represents good discrimination [40].

**Figure 2:**
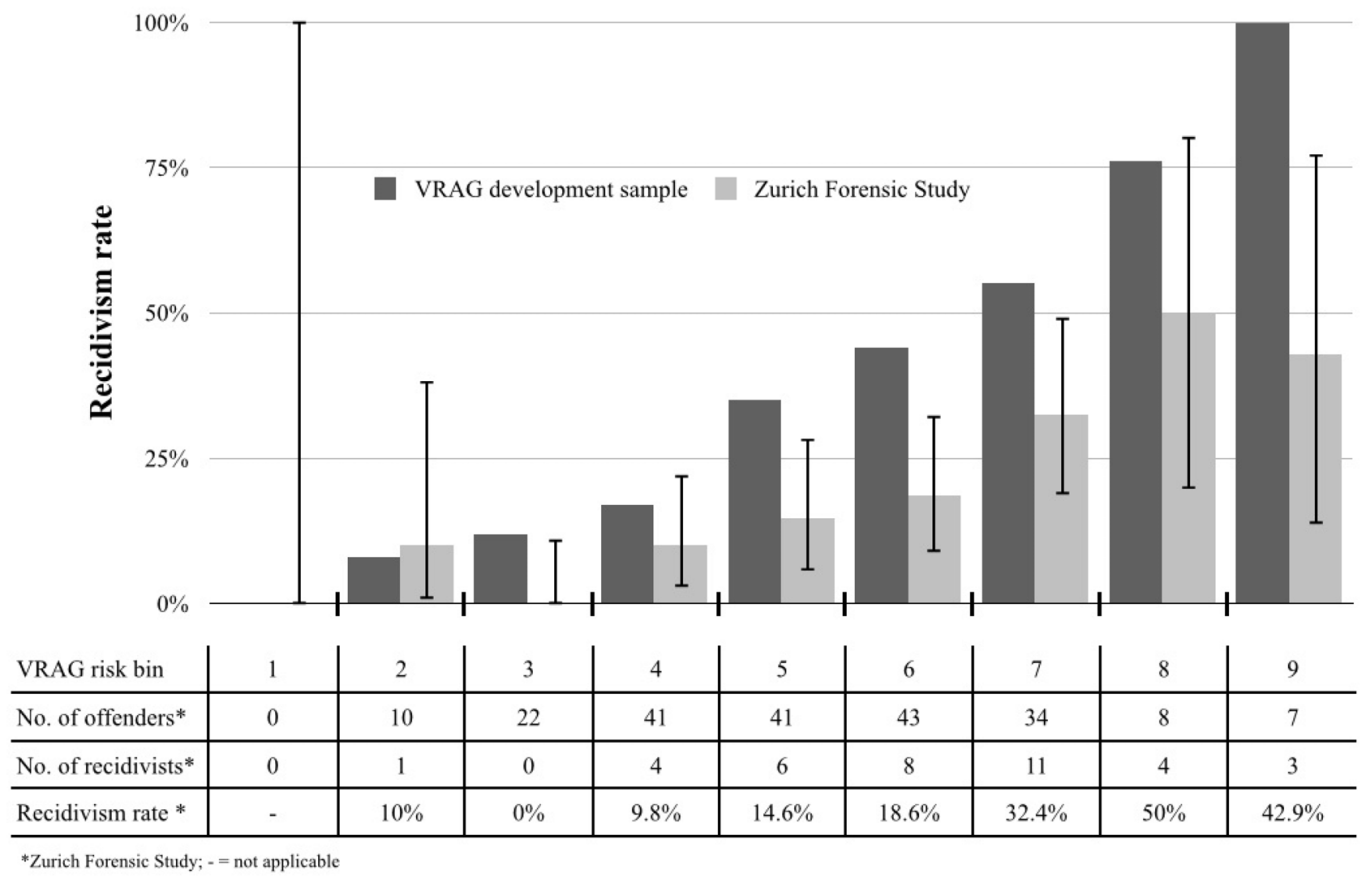
Observed 7-year post-discharge recidivism rates in the VRAG development sample and the present study including Bayesian credible intervals calculated by using the Jeffreys prior for the Beta distribution.

We explored the calibration descriptively and also analysed group differences using Sanders’ decomposition of the modified Brier score as well as the Bayesian credible intervals to investigate the significance of differences in risk rates for each VRAG risk bin (cf. Table 4). The mean VRAG score was 4.9 (*SD* = 11.7, range = −20 to +38). There were no offenders with scores warranting classification in the lowest risk bin. The majority of the offenders (*n* = 125, 60.7%) were classified in the fourth through sixth bins. The base rate of violent recidivism in the majority of the risk bins was lower in the total forensic cohort than in the VRAG development sample (Figure 2). A good overall performance of the VRAG was indicated by a Brier Score of *B* = 0.18 (AUC = 0.72). However, the Sanders’ decomposition score for the prediction of violent recidivism was 0.04, which corresponds to an average error of 21.0% per risk bin. Of particular note was the ratio of the *excess forecast variance* to the *minimum forecast variance* for the VRAG, which was 10.4. Ratios higher than 6.0 suggest substantial excess variation in risk predictions [30].

**Table 4:** Normative and observed risk bin distribution and recidivism rates for the VRAG.

VRAG risk bin	Total risk score	Percentage of sample in each risk bin		Recidivism rate (95% Bayesian credible interval^1^)	
		Harris et al. [12] 2	Zurich Forensic Study	Harris et al. [12] 2	Zurich Forensic Study
1	≤ −22	1.8%	0%	0%	- (0.00–1.00)
2	−21 to −15	11.5%	4.9%	8%	10% (0.01–0.38)
3	−14 to −8	16.3%	10.7%	12%	0% (0.00–0.11)
4	−7 to −1	18.0%	19.9%	17%	10% (0.03–0.22)
5	0 to +6	18.8%	19.9%	35%	15% (0.06–0.28)
6	+7 to +13	15.5%	20.9%	44%	19% (0.09–0.32)
7	+14 to +20	12.0%	16.5%	55%	32% (0.19–0.49)
8	+21 to +27	4.7%	3.9%	76%	50% (0.20–0.80)
9	≥ +28	1.5%	3.4%	100%	43% (0.14–0.77)

1 Bayesian credible intervals were calculated using the Jeffreys prior for the Beta distribution.

2 VRAG development sample.

In five out of nine risk bins (bins 3, 5, 6, 7, and 9), the published recidivism rates fell outside the Bayesian 95% credible interval calculated for the data from the Zurich Forensic Study and, therefore, exceeded the observed rates of recidivism (Table 4, Figure 2). This indicates a significant deviation of the published risk rates in most of the VRAG risk bins compared to those found in the current study.

## Discussion

The aim of the present study was to assess the performance of a commonly used violence risk assessment instrument, the VRAG. This research represents the first replication of the VRAG in which the dataset fulfilled the methodologic requirements of the tool’s development study including its prospective orientation, 7-year fixed length of follow-up, participant inclusion criteria, scoring protocol, and controls for sources of attrition. To ensure a comprehensive evaluation of the tool’s performance to assess violent offenders’ risk of recidivism, both discrimination (the ability to differentiate between recidivists and non-recidivists) and calibration (the fit between predicted risk and observed risk) were measured in the study.

The overall performance and discrimination validity of the VRAG was found to be good with respect to its ability to differentiate between violent recidivists and non-recidivists (*B* = 0.18 [AUC = 0.72] respectively AUC = 0.72). This level of discrimination is comparable to that reported by a number of other authors [21]. This being said, the calibration validity of the instrument was found to be poor; when we examined the observed rates of violent recidivism in each of the nine VRAG risk bins we found substantial differences compared with the expected rates as published by the tool’s developers. In addition to descriptively exploring violence rates, we also investigated calibration validity using two additional approaches: Sanders’ decomposition of the Brier score and Bayesian credible intervals for the VRAG risk bins. Using all three approaches we obtained consistent evidence that the VRAG was poorly calibrated for use in Switzerland. This corresponds to reports by other authors of poor calibration for the tool (Table 2).

### Implications

Results of the present study suggest that the VRAG lacks calibration validity. This is rather peculiar for actuarial instruments, since their key advantage over alternative approaches to risk assessment such as structured professional judgment lies in their conversion of total risk scores into probabilistic estimates of future violence risk. A poor fit of expected and observed recidivism rates limits the usefulness of actuarial risk assessment instruments in practice, because it reduces the tool’s ability to guide resource allocation and level of service classification using recidivism estimates. In legal contexts, lack of calibration validity may also lead to overestimation of the risk of future violence, resulting in long sentences, costly mandated therapy, or unnecessary community supervision. Given these serious consequences, further calibration studies using sound study protocols and comprehensive strategies for data analysis are needed. As part of this effort, the observed rates of recidivism for each risk bin should be routinely reported. Furthermore, discussion concerning the measurement of the calibration validity of risk assessment instruments needs to be advanced, as has been performed for discrimination [39]. Until this has been achieved, caution is needed when using the instrument’s probabilistic risk estimates in practice.

In accordance with a Bayesian approach, recent meta-analyses of literature on risk assessment for both violent [41] and sex [42], [43] offenders suggest that it might not be possible to reliably assign an expected probability to a group without taking into consideration population-based priors. This raises the following question: if the published expected recidivism rates for the nine VRAG risk bins are not reliable, of what practical use are differences between bins? For example, what actions would be appropriate for the individuals in bin 4 that would not be needed for individuals in bin 3?

Previous studies have endeavoured to measure the calibration validity of the VRAG using either the *χ* ^2^ goodness-of-fit index or the correlation coefficient, both of which have limited usefulness for this task. Regarding the former, the goodness-of-fit index is calculated using the expected rate of violence as specified in the VRAG manual and the rate of violence observed in a given replication study:
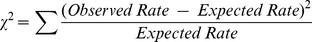



The first notable issue when using this calibration parameter with the VRAG is that the expected rate of violence for the lowest risk bin of the instrument is 0%, resulting in division by zero. Adding a small constant in instances of zero cell counts allows non-parametric analyses to proceed [44] but can result in considerably biased χ^2^ estimates. For example, Harris and colleagues [45] found that 17% of individuals in the lowest risk bin of the VRAG went on to violently recidivate. Using a substitute of 1% for the expected rate results in a single risk bin χ^2^ of 256, well above the α = 0.05 critical threshold of 15.51. A second limitation of the index is that differences in expected and observed rates of violence in lower risk bins have a larger influence on the resulting *χ* ^2^ estimate than differences in higher bins. For example, a deviation of 5% in the highest risk bin has almost no statistical influence, whereas the same 5% mismatch in the lowest risk bin will have considerable impact. A third obstacle that needs to be considered when using the goodness-of-fit test is that risk assessment tools developed in populations with higher base rates will have poorer calibration estimates when replicated in samples with higher rates of violence than in those with lower rates. Given the substantial variability in the rates of violence in VRAG studies [46], this may be an important issue to consider in some studies. For these reasons, goodness-of-fit tests may be inappropriate for measuring calibration validity in replication studies whose samples are derived from populations with higher overall base rates (or at least higher rates of violence in individuals with lower VRAG scores). The correlation coefficient (*r*) is similarly limited in that deviations of even 20% in the expected rate of violence in each risk bin can still produce statistically significant evidence of good calibration.

### Limitations

The present sample represented a total forensic cohort in Switzerland, a country with a criminal justice system based on civil law. The VRAG, however, was developed in a common law jurisdiction (Ontario, Canada), meaning that several items are couched in jurisprudence that is not relevant abroad. Thus, it is perhaps understandable that the VRAG performed poorly in terms of calibration validity in our investigation despite our attempts to replicate the conditions of the instrument’s development study as closely as possible. This said, the authors of the VRAG manual have previously claimed that the tool can be used in international settings based on discrimination findings using performance indicators such as the AUC and correlation coefficient. Taking into consideration the present findings together with previous reports that the instrument’s probabilistic estimates of future violence risk do not hold in other countries including Germany, Sweden, the United Kingdom, and the United States, the developers of actuarial risk assessment tools might need to revise their conclusions concerning generalizability. One way forward could be the establishment and incorporation of jurisdiction-specific norms for group-based risk estimates, which would allow for greater cultural sensitivity when instruments developed in one country are implemented in another.

### Conclusion

The performance of violence risk assessment tools has two components: discrimination and calibration. To date, studies have primarily focused on discrimination, and calibration has been largely neglected. However, both components need to be established before concluding that a risk assessment instrument is useful in practice. The large body of discrimination evidence for actuarial instruments such as the VRAG belies scant calibration findings that suggest poor performance in prospective risk assessment using probabilistic risk estimates. In the end, although the performance of the instrument with respect to discrimination indicates potential of the VRAG, its poor calibration results raise questions regarding its practical usefulness.

## References

[1] Steadman HJ, Cocozza JJ (1974) Careers of the criminally insane: Exzessive social control of deviance. Lexington: Lexington Books. 227 p.

[2] Thornberry T, Jacoby J (1979) The criminally insane: A community follow-up of mentally ill offenders. T. Chicago: University of Chicago Press. 299 p.

[3] Association AP (1974) Task force report 8: Clinical aspects of the violent individual. Washington, DC: American Psychiatric Association.

[4] Association AP (1978) Task force on ect: Electroconvulsive therapy, task force report #14. Washington, DC: American Psychiatric Association.

[5] Monahan J (1981) Predicting violent behavior: An assessment of clinical techniques. Beverly Hills: CA: Sage.

[6] Grove WM, Zald DH, Lebow BS, Snitz BE, Nelson C (2000) Clinical versus mechanical prediction: A meta-analysis. Psychological Assessment 12: 19–30. 10752360

[7] Swets JA, Dawes RM, Monahan J (2000) Better decisions through science. American Scientific 283: 82–87. 10.1038/scientificamerican1000-82 11011389

[8] Hanson RK, Howard PD (2010) Individual confidence intervals do not inform decision-makers about the accuracy of risk assessment evaluations. Law and Human Behavior 34: 275–281. 20556495 10.1007/s10979-010-9227-3

[9] Archer RP, Buffington-Vollum JK, Stredny RV, Handel RW (2006) A survey of psychological test use patterns among forensic psychologists. Journal of Personality Assessment 87: 84–94. 16856789 10.1207/s15327752jpa8701_07

[10] Viljoen JL, McLachlan K, Vincent GM (2010) Assessing violence risk and psychopathy in juvenile and adult offenders: A survey of clinical practices. Assessment 17: 377–395. 20124429 10.1177/1073191109359587

[11] Quinsey VL, Harris GT, Rice ME, Cormier CA (2006) Violent offenders: Appraising and managing risk. Washington DC: American Psychological Association. 462 p.

[12] Harris GT, Hilton NZ, Rice ME (1993) Patients admitted to psychiatric hospital: Presenting problems and resolution at discharge. Canadian Journal of Behavioural Science 25: 267–285.

[13] Nuffield J (1982) Parole decision-making in Canada: Research towards decision guidelines. Ottawa: Solicitor General of Canada.

[14] Fazel S, Singh JP, Doll H, Grann M (2012) The prediction of violence and antisocial behaviour: A systematic review and meta-analysis of the utility of risk assessment instruments in 73 samples involving 24,827 individuals. British Medical Journal.10.1136/bmj.e4692. 10.1136/bmj.e4692 PMC3404183 22833604

[15] Rossegger A, Gerth J, Seewald K, Urbaniok F, Singh JP, et al (2013) Current obstacles in replicating risk assessment findings: A systematic review of commonly used actuarial instruments. Behavioral Sciences and the Law 31: 154–164. 23408438 10.1002/bsl.2044

[16] Singh JP (2013) Predictive validity performance indicators in violence risk assessment: A methodological primer. Behavioral Sciences & the Law 31: 8–22. 23408459 10.1002/bsl.2052

[17] Donaldson T, Wollert R (2008) A mathematical proof and example that Bayes’s theorem is fundamental to actuarial estimates of sexual recidivism risk. Sexual Abuse: A Journal of Research and Treatment 20: 206–217. 18490482 10.1177/1079063208317734

[18] Hart SD, Michie C, Cooke DJ (2007) Precision of actuarial risk assessment instruments: Evaluating the ‘margins of error’ of group v. individual predictions of violence. British Journal of Psychiatry 49: 60–65. 10.1192/bjp.190.5.s60 17470944

[19] Mossman D (2006) Another look at interpreting risk categories. Sexual Abuse: A Journal of Research and Treatment 18: 41–63. 16639536 10.1177/107906320601800104

[20] Urbaniok F, Rinne T, Held L, Rossegger A, Endrass J (2008) Forensic risk calculation: Basic methodological aspects for the evaluation of the applicability and validity of diverse methods. Fortschritte der Neurologie und Psychiatrie 76: 470–477. 10.1055/s-2008-1038228 18677678

[21] Waypoint (2012) Penetanguishene, ON: Waypoint Centre for Mental Health Care.

[22] Endrass J, Rossegger A, Urbaniok F (2007) Zürcher Forensik Studie, Abschlussbericht eines Modellversuchs: Therapieevaluation und Prädiktorenforschung. Zurich: Criminal Justice System, Canton of Zurich. 175p.

[23] Hare RD (2003) Manual for the Revised Psychopathy Checklist. Toronto ON: Multi-Health Systems.

[24] Rossegger A, Urbaniok F, Danielsson C, Endrass J (2009) The Violence Risk Appraisal Guide (VRAG) - a tool for the risk assessment of violent offenders. Fortschritte der Neurologie und Psychiatrie 77: 577–584. 10.1055/s-0028-1109705 19821220

[25] Fleiss JL, Levin B, Paik MC (2004) The measurement of interrater agreement. In: Statistical methods for rates and proportions 3 ed. Hoboken, New York: John Wiley & Sons. doi: 10.1002/0471445428.ch18.

[26] Schmid CH, Griffith JL (2005) Multivariate classification rules: Calibration and discrimination. In: Armitage P, Colton T, editors. Encyclopedia of biostatistics. 2 ed: John Wiley & Sons,10.1002/0470011815.b2a13049.

[27] Vergouwe Y, Steyerberg EW, Eijkemans MJC, Habbema JDF (2005) Substantial effective sample sizes were required for external validation studies of predictive logistic regression models. Journal of Clinical Epidemiology 58: 475–483. 15845334 10.1016/j.jclinepi.2004.06.017

[28] Rufibach K (2010) Use of Brier score to assess binary predictions. Journal of Clinical Epidemiology 63: 938–942. 20189763 10.1016/j.jclinepi.2009.11.009

[29] Steyerberg EW, Vickers AJ, Cook NR, Gerds T, Gonen M, et al (2010) Assessing the performance of prediction models - a framework for traditional and novel measures. Epidemiology 21: 128–138. 20010215 10.1097/EDE.0b013e3181c30fb2 PMC3575184

[30] Spiegelhalter D (1986) Probabilistic prediction in patient management and clinical trials. Statistics in Medicine 5: 421–433. 3786996 10.1002/sim.4780050506

[31] Rogers W (1992) Brier score decomposition. Stata Technical Bulletin: 20–22.

[32] Ferro CAT (2007) Comparing probabilistic forecasting systems with the Brier score. Weather & Forecasting 22: 1076–1088.

[33] Redelmeier D, Bloch D, Hickam D (1991) Assessing predictive accuracy: How to compare Brier scores. Journal of Clinical Epidemiology 44: 1141–1146. 1941009 10.1016/0895-4356(91)90146-z

[34] Kass RE, Wasserman L (1996) The selection of prior distributions by formal rules Journal of the American Statistical Association. 91: 1343–1370.

[35] Breslow N (1990) Biostatistics and Bayes. Statistical Science 5: 269–298.

[36] Carlin BP, Louis TA (2009) Approaches to statistical inferences. In: Bayesian methods for data analysis 3 ed. Boca Raton, FL: CRC Press.

[37] Edwards W, Lindman H, Savag LJ (1963) Bayesian statistical inference for psychological research. Psychological Review 70: 193–242.

[38] StataCorp (2012) Stata statistical software: Release 12. College Station: TX: StataCorp LP.

[39] Singh JP, Desmarais S, Van Dorn RA (2013) Measurement of predictive validity in violence risk assessment studies: A second-order systematic review. Behavioral Sciences & the Law 31: 55–73. 23444299 10.1002/bsl.2053

[40] Rice ME, Harris GT (2005) Comparing effect sizes in follow-up studies: Roc area, cohen’s d, and r. Law and Human Behavior 29: 615–620. 16254746 10.1007/s10979-005-6832-7

[41] Singh JP, Fazel S, Gueorguieva R, Buchanan A (2014) Rates of violence in patients classified as “high risk” by risk assessment instruments. British Journal of Psychiatry. (in press). 10.1192/bjp.bp.113.131938 PMC3939440 24590974

[42] Helmus L, Hanson RK, Thornton D, Babchishin KM, Harris AJR (2012) Absolute recidivism rates predicted by Static-99R and static-2002R sex offender risk assessment tools vary across samples: A meta-analysis. Criminal Justice and Behavior 39: 1148–1171.

[43] Singh JP, Fazel S, Gueorguieva R, Buchanan A (2013) Rates of sexual recidivism in high risk sex offenders: A meta-analysis of 10,422 participants. Sexual Offender Treatment 7: 44–57.

[44] Higgins J, Deeks J, Altman DG (2008) Special topics in statistics. In: Higgins J, Green S, editors. Cochrane handbook for systematic reviews of interventions 500. London: John Wiley & Sons. pp. 481–529.

[45] Harris GT, Rice ME, Cormier CA (2002) Prospective replication of the Violence Risk Appraisal Guide in predicting violent recidivism among forensic patients. Law and Human Behavior 26: 377–394. 12182529 10.1023/a:1016347320889

[46] Singh JP, Fazel S, Gueorguieva R, Buchanan AR (2013) Rates of sexual recidivism in high risk sex offenders: A meta-analysis of 10,422 participants. Sexual Offender Treatment 7: 44–57.

[47] Tengström A (2001) Long-term predictive validity of historical factors in two risk assessment instruments in a group of violent offenders with schizophrenia. Nordic Journal of Psychiatry 55: 243–249. 11839114 10.1080/080394801681019093

[48] Harris GT, Rice ME, Quinsey VL, Lalumière ML, Boer D, et al (2003) A multisite comparison of actuarial risk instruments for sex offenders. Psychological Assessment 15: 413–425. 14593842 10.1037/1040-3590.15.3.413

[49] Mills JF (2005) An examination of the generalizability of the LSI-R and VRAG probability bins. Criminal Justice and Behavior 32: 565–585.

[50] Yessine AK, Bonta J (2006) Tracking high-risk, violent offenders: An examination of the national flagging system. Canadian Journal of Criminology and Criminal Justice 48: 573–607.

[51] Snowden RJ, Gray NS, Taylor J, MacCulloch MJ (2007) Actuarial prediction of violent recidivism in mentally disordered offenders. Psychological Medicine 37: 1539–1549. 17537287 10.1017/S0033291707000876

[52] Kröner C, Stadtland C, Eidt M, Nedopil N (2007) The validity of the Violence Risk Appraisal Guide (VRAG) in predicting criminal recidivism. Criminal Behaviour and Mental Health 17: 89–100. 17295202 10.1002/cbm.644

[53] Hastings ME, Krishnan S, Tangney JP, Stuewig J (2011) Supplemental material for predictive and incremental validity of the Violence Risk Appraisal Guide scores with male and female jail inmates. Psychological Assessment. 10.1037/a0021290 PMC3074300 21381844

